# Fabrication of Patterned Magnetic Particles in Microchannels and Their Application in Micromixers

**DOI:** 10.3390/bios14090408

**Published:** 2024-08-23

**Authors:** Tianhao Li, Chen Yang, Zihao Shao, Ya Chen, Jiahui Zheng, Jun Yang, Ning Hu

**Affiliations:** Key Laboratory of Biorheological Science and Technology, Ministry of Education and Bioengineering College, Chongqing University, Chongqing 400044, China; tianhaoli@stu.cqu.edu.cn (T.L.); yangchencq@cqu.edu.cn (C.Y.); spoonzh@stu.cqu.edu.cn (Z.S.); 202319131165@stu.cqu.edu.cn (Y.C.); jiahuizheng@cqu.edu.cn (J.Z.)

**Keywords:** microfluidic chip, particle fabrication device, micromixer, magnetic particles

## Abstract

Due to the extremely low Reynolds number, the mixing of substances in laminar flow within microfluidic channels primarily relies on slow intermolecular diffusion, whereas various rapid reaction and detection requirements in lab-on-a-chip applications often necessitate the efficient mixing of fluids within short distances. This paper presents a magnetic pillar-shaped particle fabrication device capable of producing particles with planar shapes, which are then utilized to achieve the rapid mixing of multiple fluids within microchannels. During the particle fabrication process, a degassed PDMS chip provides self-priming capabilities, drawing in a UV-curable adhesive-containing magnetic powder and distributing it into distinct microwell structures. Subsequently, an external magnetic field is applied, and the chip is exposed to UV light, enabling the mass production of particles with specific magnetic properties through photo-curing. Without the need for external pumping, this chip-based device can fabricate hundreds of magnetic particles in less than 10 min. In contrast to most particle fabrication methods, the degassed PDMS approach enables self-priming and precise dispensing, allowing for precise control over particle shape and size. The fabricated dual-layer magnetic particles, featuring fan-shaped blades and disk-like structures, are placed within micromixing channels. By manipulating the magnetic field, the particles are driven into motion, altering the flow patterns to achieve fluid mixing. Under conditions where the Reynolds number in the chip ranges from 0.1 to 0.9, the mixing index for substances in aqueous solutions exceeds 0.9. In addition, experimental analyses of mixing efficiency for fluids with different viscosities, including 25 wt% and 50 wt% glycerol, reveal mixing indices exceeding 0.85, demonstrating the broad applicability of micromixers based on the rapid rotation of magnetic particles.

## 1. Introduction

The fluid flow in microfluidic systems usually exhibits a very low Reynolds number (Re), which is characteristic of laminar flow. In laminar flow conditions, the mixing of substances between different flow layers is primarily achieved through molecular diffusion, which is a process that is inefficient and slow [1,2,3]. Applications such as rapid detection, on the other hand, place high demands on the mixing of various reactants. It is therefore crucial to achieve rapid and thorough mixing over short distances within microfluidic systems. Presently, micromixers in microfluidic systems can be broadly categorized into two types: active mixers, which are driven by external energy sources, and passive mixers, which do not require external energy input.

Passive mixers are typically constructed with intricate or elongated channels in order to enhance mixing efficiency. As an illustration, serpentine channels [4,5,6,7] and herringbone channels [8] can increase the interfacial area, and T-channels with non-aligned inputs can create vortexes within the channel [9]. In addition, some researchers have placed impellers [10] or ablated irregular microspheres [11] within the channel to reduce the channel length for complete mixing. Passive mixers facilitate mixing without requiring external energy input. However, their intricate channels and structures limit their usage on smaller microfluidic chips. Additionally, there is still room for improvement in the effectiveness of passive mixing at extremely low Reynolds numbers [12,13]. Active mixers in microfluidic systems utilize external energy to achieve fluid mixing. For example, acoustic fields can be used to generate acoustic vortices that control fluid mixing [14,15,16]. Similarly, an external magnetic field is applied to enhance the mixing of microchannels or microdroplets [17,18]. Additionally, small bubbles can pass through the channels to serve as an active mixer for fluid mixing [19].

One interesting option for active mixing is the production of multifunctional magnetic particles that can rotate under a magnetic field, thereby stirring the fluid. Magnetic force, as a biocompatible and non-contact force, has been extensively utilized in microfluidic devices [20,21]. By utilizing magnetic particles as stirring rods, rapid and thorough mixing can be achieved over short distances, yielding exceptional mixing outcomes even at extremely low Reynolds numbers [22]. Most of the magnetic particles employed in current research were shown to adopt relatively uniform shapes, predominantly rod-shaped [18,22,23,24,25]. In practice, experimenting with magnetic particles of various configurations can be attempted to enhance mixing efficiency and adaptability.

Microfluidics, as an emerging technology, has been widely used in biochemical analysis and detection [26,27,28,29,30], as well as drug extraction and enrichment [31,32,33]. The utilization of microfluidics to fabricate droplets and particles, as well as the application of these fabricated particles in microscale systems, has consistently been a popular research area [34,35,36]. Microfluidic chips, for example, can generate spherical particles with adjustable scale and high dispersion by controlling the flow rate and optimizing structural parameters at the microscale [37,38]. Spherical or rod-shaped particles with different physical properties can be fabricated using fluid self-assembly and the application of light [39,40,41]. Microfluidic devices for particle manufacture offer benefits such as rapid fabrication, portability, low cost, and high output efficiency.

This work involves the creation of a device that distributes samples into patterned microstructures through negative pressure to produce magnetic particles. Using this device, various shapes of magnetic particles have been designed and fabricated, serving as stirring elements for active mixers to address the challenge of insufficient mixing efficiency within microchannels. By utilizing a degassed PDMS chip as a self-priming source, the UV-curable adhesive-containing magnetic powder can be successfully sucked into the chip without the need for an external pumping component. By adjusting the microwell structure within the chip, the shape, size, and height of the magnetic particles can be controlled. Experimental results demonstrate that, driven by a magnetic field, the magnetic particles can rotate within microchannels, thereby generating significant disturbances to the laminar flow at low Reynolds numbers and effectively mixing various color dye solutions, including even high-viscosity glycerol solutions. In addition, multiple particles can be placed simultaneously in the microchannel, and their rotation can be controlled individually. Since these particle movements require only an extremely short startup time (within 4 s), they enable mixing missions at specific locations within the microchannel at a designated time.

## 2. Experimental Section

### 2.1. Design of Microfluidic Chips

This study describes a chip composed of two layers of PDMS slabs and a piece of glass slide with identical lengths and widths. The chip measures 25 mm in length, 18 mm in width, and 6.6 mm in height. It features a liquid inlet and four liquid outlets on the chip, which are interconnected by four channels to form a flow network for the directional flow of sample fluids within the chip. To enhance the production of microparticles, 336 microwells have been designed into each chip, which are evenly distributed on both sides of the channel. To facilitate the smooth entry of the UV-curable adhesive into the microwells, the inlet of the chip has been expanded and formed into a hollow cylindrical structure with a radius of 700 microns. To ensure the smooth rotation of particles in the microchannel, the channel that holds the magnetic particles for rotational stirring has been designed with a height of 525 microns and a radius of 560 microns.

Unlike most microfluidic structures that produce rod-shaped or spherical particles, the microwells in this research are designed with a bilayer structure, allowing for the creation of particles with more complex three-dimensional geometries and imparting different functions to each layer. Specifically, the lower layer of the microparticle structure has been designed in the shape of a fan blade for mixing fluids, while the upper layer takes the form of a disk, which facilitates smoother rotation of the particles. During the course of self-dispensing, air unavoidably removes a small amount of the UV-curable adhesive, compromising the structural integrity of the particles. To overcome this issue, the microwells have been designed such that only the upper layer is connected to the feed channel, ensuring a high level of structural integrity for the more essential lower layer.

### 2.2. Fabrication of Chips and UV-Curable Composites

**Figure 1:**
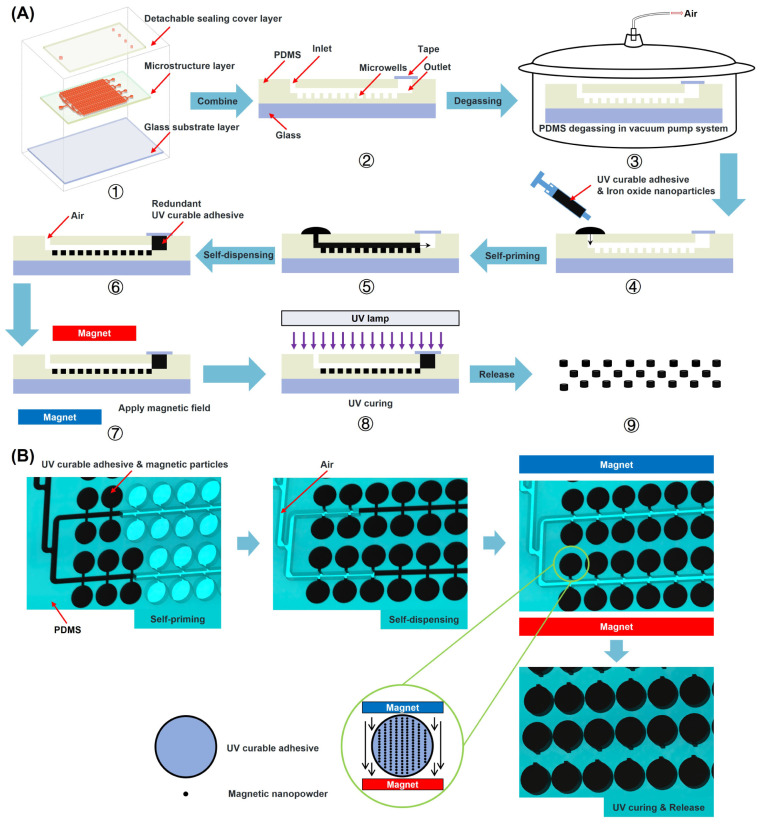
Schematic of the chip design and particle fabrication mechanism. (**A**) Schematic of the microfluidic chip and the process of manufacturing particles: ① The microfluidic chip consists of two layers of PDMS and a glass substrate layer. ② Combine the three layers together and tape the outlet. ③ The PDMS chip undergoes degassing in a vacuum pump system. ④ Take out the PDMS chip and add drops of mixed solution to the inlet. ⑤ and ⑥ The process of self-priming and self-dispensing. ⑦ and ⑧ Apply magnetic field and UV light to the chip. ⑨ Take out the detachable sealing cover layer and release the particles. (**B**) A 3D schematic of the in-chip particle manufacturing process.

The multilayer soft lithography technique was employed to fabricate the microfluidic chips. Firstly, the photo mask was created using AutoCAD 2022 software (San Rafael, CA, USA), and a master mold was then produced on a 4-inch silicon wafer through photolithography processing using photoresist (SU8-2050). A master mold containing a two-layer structure was fabricated using double photolithography, which gave the chip the ability to fabricate two-layer particles [as indicated in Figure S1]. Subsequently, the patterns on the silicon master were transferred onto a PDMS chip as the microstructured layer using the replica molding method, enabling the manufacture of microwell arrays with different structures. A detachable sealing cover layer was tightly affixed onto the microstructured layer, which was then horizontally placed on the glass surface and sealed at the four fluid outlets with tape (Figure 1A); thus, the chip fabrication was completed.

LOCTITE AA 3311 is a colorless and transparent medical-grade UV-curable adhesive with outstanding biocompatibility, which is widely employed in various fields, including medical syringes and catheter components. In this study, LOCTITE AA 3311 was utilized to formulate a suspension of Fe_3_O_4_ nanoparticles (magnetic powder) for the purpose of manufacturing magnetic particles. Specifically, during the fabrication process, a measured amount of magnetic powder [as indicated in Table S1] was mixed with the UV-curable adhesive in a centrifuge tube. Subsequently, the centrifuge tube was placed in a vortex mixer to thoroughly blend the magnetic powder with the adhesive. This suspension possesses excellent fluidity and is capable of rapid curing upon exposure to UV light.

### 2.3. The Manufacturing Process of Particles

Air was utilized instead of oil to remove excess suspension in the main channel, thereby achieving optimized self-dispensing technology. Compared to oil, air has a much lower viscosity, enabling mixed suspension within the chip to complete the self-dispensing process at a considerably higher rate. Meanwhile, employing air to separate the mixed suspension guarantees that it remains uncontaminated by oil, thus enhancing the fidelity of the fabricated particles. Using this technique, the mixed suspension can be evenly distributed into 336 microwells on the chip within just 10 min.

Figure 1A illustrates the manufacturing process of particles: Initially, the PDMS chip was placed in a vacuum pump system (Vi-120SA, Zhejiang, China) for degassing, maintaining a pressure of −1 Kpa for a duration of 3 h. Due to the permeable and porous characteristics of PDMS, the internal cavity structure of the chip can maintain a negative pressure for an extended period even after being removed from the vacuum pump. Following this, the chip was taken out of the vacuum pump, and the mixed suspension was dripped onto the fluid inlet. Subsequently, the mixed suspension was automatically drawn into the microstructure layer of the chip.

Once the mixed suspension had completely filled the entire chamber, excess liquid at the inlet of the cover layer was wiped off to allow air to enter the microstructured layer through the inlet. The airflow will then propel the mixed suspension within the microchannels towards the four outlets of the chip, without affecting the suspension that has already settled into the microwells. Consequently, the airflow can separate the microwells into individually filled entities and expel excess liquid through the chip outlets.

**Figure 2:**
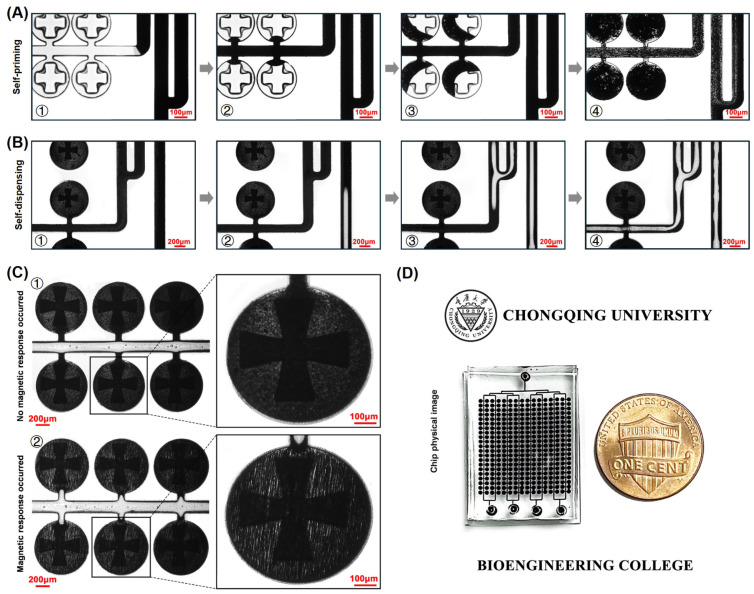
Physical diagram of the particle manufacturing process and the chip. (**A**) Degassed PDMS fills the microwells with mixed solution by self-priming: ① and ② Mixed solution filled microchannels. ③ and ④ Mixed solution filled microwells. (**B**) Air enters the main channel to push away the excess mixed solution: ① and ② Air filled microchannels. ③ and ④ Air pushed away the excess mixed solution. (**C**) Magnetic field applied to the chip: ① Before the magnetic field is applied, the nanomagnetic powders are uniformly distributed. ② After the magnetic field is applied, the nanopowders are magnetically responsive and aligned in chains along the direction of the field. (**D**) A physical image of the chip used to prepare the particles.

After the mixed suspension was separated into distinct entities by air, the opposite poles of two magnets were positioned on both sides of the chip and held in place for a duration of 20 s (Figure 1B). The purpose of this step is to induce a magnetic response in the magnetic powder, which will be attracted by the magnetic field and organized into a structure resembling a chain, being easily observable under a microscope (Figure 2C). Following this, the chip was thereafter exposed to a UV lamp in order to solidify the mixed suspension within the microwells. After the particle curing process, the top cover slide was removed. We poured water over the microstructured layer of the mold and then froze it in a freezer at −10 °C for 70 min. Subsequently, microparticles incorporating ice were detected from the mold template. By removing the ice and waiting for it to melt, a solution with particles was collected.

## 3. Results and Discussion

### 3.1. Manufacturing Evaluation of Magnetic Particles

**Figure 3:**
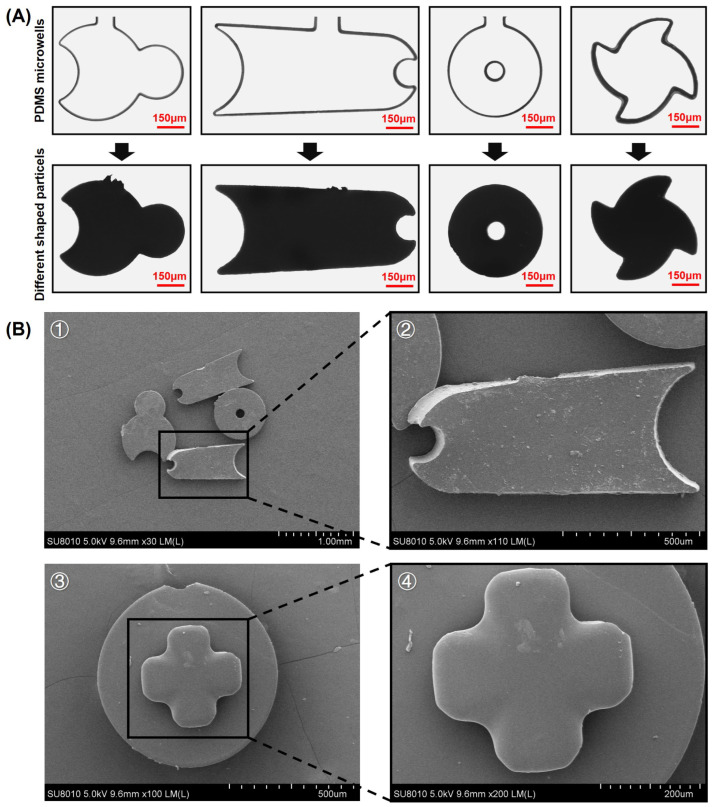
Physical and SEM images of particles. (**A**) PDMS microwells used to produce particles, as well as various shapes of microwell-fabricated particles. (**B**) SEM images of particles: ① and ② Particles manufactured in various shapes, demonstrating that even complicated structures exhibit outstanding fidelity in the plane. ③ and ④ Disk-like bilayer particle with fan-shaped blades, demonstrating that the particles have high fidelity and a smooth surface.

The particles fabricated by the approach of self-priming and air separation exhibit the advantage of high fidelity. As shown in Figure 3A, particles of various shapes have been fabricated. The first row in Figure 3A depicts the PDMS microwells, and the second row displays the particles fabricated by using the corresponding microwells in the first row. It can be observed that the fabricated particles almost perfectly mirror the shapes of the microwells, indicating a very high level of fidelity. Figure 3B presents SEM images of various particles, showcasing not only their high fidelity but also the extremely smooth and well-organized three-dimensional structure of the multilayer particles.

Figure 3B③ depicts the three-dimensional structure of a bilayer particle. The upper layer of the particle is the more important fan-blade portion, which serves the function of agitating the fluid; the lower layer of the particle is the disk-shaped substrate, which facilitates the rotation of the particle within the chamber without getting stuck. During the air separating process, a small amount of UV-curable adhesive is carried away by the air, resulting in a slight loss of shape in the lower layer of the particle. However, due to the unique design of the microwells, the more important upper layer maintained a near-perfect shape during the air splitting process. The high fidelity of the fan-shaped layer ensures the stability of the subsequent mixing efficiency, while the disc-like structure enables the smooth rotation of the particle even with minor shape loss.

### 3.2. Optimization of Particle Output Efficiency and Particle Rotation

**Figure 4:**
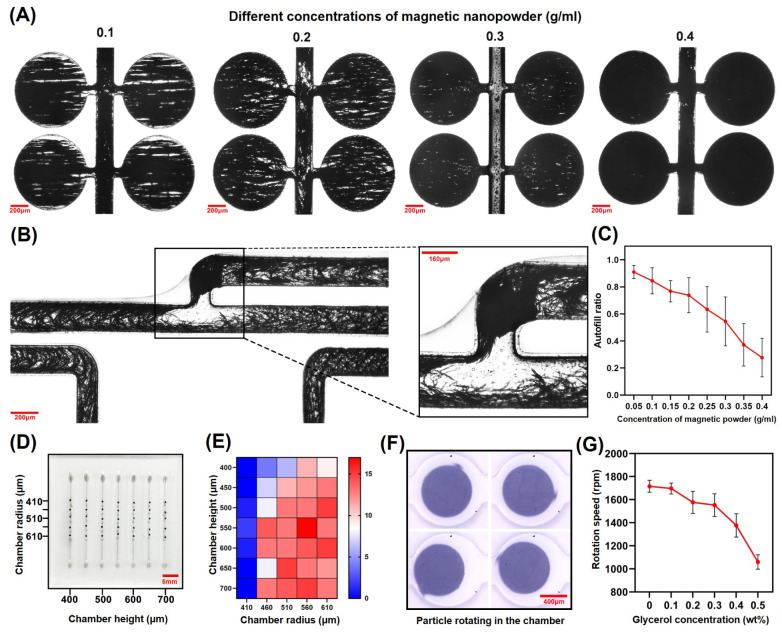
Optimization of particulate output efficiency and analysis of particulate rotation. (**A**) UV-curable adhesive doped with different concentrations of magnetic nanopowder enters the chambers through self-priming. (**B**) When the concentration of magnetic nanopowder in the UV-curable adhesive is high, it may lead to the blockage of the main channel by the nanopowder, which may reduce the output efficiency of the particles. (**C**) The effect of different concentrations of magnetic powder on the particle output efficiency (each concentration was repeated 5 times). (**D**) PDMS chip used to investigate the rotation of particles in chambers of different sizes. (**E**) Statistical graph of particle rotation in different chambers. (**F**) Using a high-speed camera to record the rotation speed of particles inside the chamber. (**G**) Statistical plot of rotational speed of particles in different concentrations of glycerol (each concentration was repeated 10 times).

In this study, a higher concentration of magnetic powder in the particles that enhanced the response of the particles to the external magnetic field was observed, which allowed the particles to continue to rotate under viscous fluids. However, a higher concentration of magnetic powder would block the channels of the microstructured layer (Figure 4B) and induce the particle output efficiency to decrease. Conversely, lower concentrations of magnetic powder led to particles with poor magnetic properties, being unable to promptly respond to the external rotating magnetic field. This prevented the particles from rotating and completing the subsequent mixing tasks. Consequently, mixed suspensions of Fe_3_O_4_ nanoparticles with concentrations of 0.05 g/mL, 0.1 g/mL, 0.15 g/mL, 0.2 g/mL, 0.25 g/mL, 0.3 g/mL, 0.35 g/mL, and 0.4 g/mL were fabricated, and we used these for the manufacturing of the particles and for recording the output efficiency (Figure 4C). It is clear that the suspension containing 20 wt% Fe_3_O_4_ powder (0.2 g/mL) exhibited a good output efficiency, and the particles created at this concentration demonstrated a prompt response to the external rotating magnetic field. Therefore, this suspension ratio was adopted for subsequent mixing trials. In addition, the use of refined magnetic powder can further increase the efficiency of particle output [as indicated in Figure S4]. Therefore, magnetic powder with a particle size of 20 nm was used for the preparation of particles in this study.

If the magnetic particles rotate unevenly in chambers of varying heights and radii, this may lead to inconsistent mixing effects when using these magnetic particles as stirrers. To ensure stable and reproducible mixing efficiency, a PDMS chip containing 35 cylindrical chambers was developed (Figure 4D). These chambers were distributed in seven columns and five rows. Each column features the same height, while the chamber radius gradually increases from 410 to 610 µm. Alternatively, in another design iteration, the radii of the chambers within each row remained constant, while their heights varied from 400 to 700 µm. Particles were placed within each chamber, and a rotating magnetic field was applied to the bottom of the chip. The rotation of particles within each chamber was then counted (Figure 4E). The results of 20 replications indicated that the particles exhibited better rotational behavior when the volume ratio of particles to chambers fell between 0.2 and 0.3. Therefore, incorporating our machining process, the radius and height of the cylindrical chamber were set to 560 µm and 525 µm, respectively, in the next study.

A high-speed camera (Phantom Veo 710L, Dey Road Wayne, NJ, USA) was used to record the rotation process of particles in water and glycerol amounts of different concentrations (Figure 4F). As can be observed from Figure 4G, when the same rotating magnetic field was applied, the rotation speeds of the particles in different fluid media reduced with the increase in viscosity.

### 3.3. Magnetic Particles as Stirrers for Active Mixing

Since the magnetic powder within the particles can respond to an applied magnetic field, magnetic particles can be manipulated using magnetic fields to achieve behaviors such as moving, flipping, and rotating, thereby endowing these magnetic particles with the potential to construct mixers. To test the mixing performance of magnetic particles, a Y-shaped PDMS channel for mixing experiments was fabricated. It was composed of two fluid inlets, one outlet, and a cylindrical chamber for the particle rotation. The fluid channel has a width of 400 μm (slightly smaller than the particle size, with a radius of 360 μm) and a height of 525 μm, while the cylindrical chamber has a radius of 560 μm (slightly larger than the particles). This design allows magnetic particles to rotate precisely within the cylindrical chamber without entering the fluidic channels.

To investigate the mixing, magnetic particles were placed within the cylindrical chamber (using tweezers to place particles in the mixing channel), and the Y-shaped PDMS channel was bonded to the glass substrate. Clear water and blue dye ink were chosen as the experimental samples, and two conduits were installed at the two fluid inlets to input the water and dye ink at a specific flow rate while maintaining both of the fluid phases in laminar flow. After passing through the cylindrical chamber, without the particle rotation, there was no significant mixing observed between the two fluid phases. However, when a rotating magnetic field was applied beneath the Y-shaped PDMS channel, the magnetic particles were observed to rotate constantly, acting as micromixers for the two-phase fluids. A microscope and a camera were used to capture the mixing process, and color information was extracted at four locations within the 5.4 mm long microchannel. The mixing indices (MIs) were calculated under various conditions to assess the mixing effectiveness.

In order to investigate the mixing efficiency of particles in high-viscosity fluids, 50 wt% glycerol containing red dye ink and colorless 50 wt% glycerol were used as samples for mixing experiments. Glycerol was introduced into the Y-shaped PDMS channel, and the same method was utilized to quantify the mixing results. When a rotating magnetic field was applied, the particles were able to rotate in the glycerol solution, accelerating the mixing of the two glycerol solutions through agitation. This demonstrated that particles could accomplish the mixing task in systems with higher viscosity.

### 3.4. Evaluation of Particle Mixing Performance

**Figure 5:**
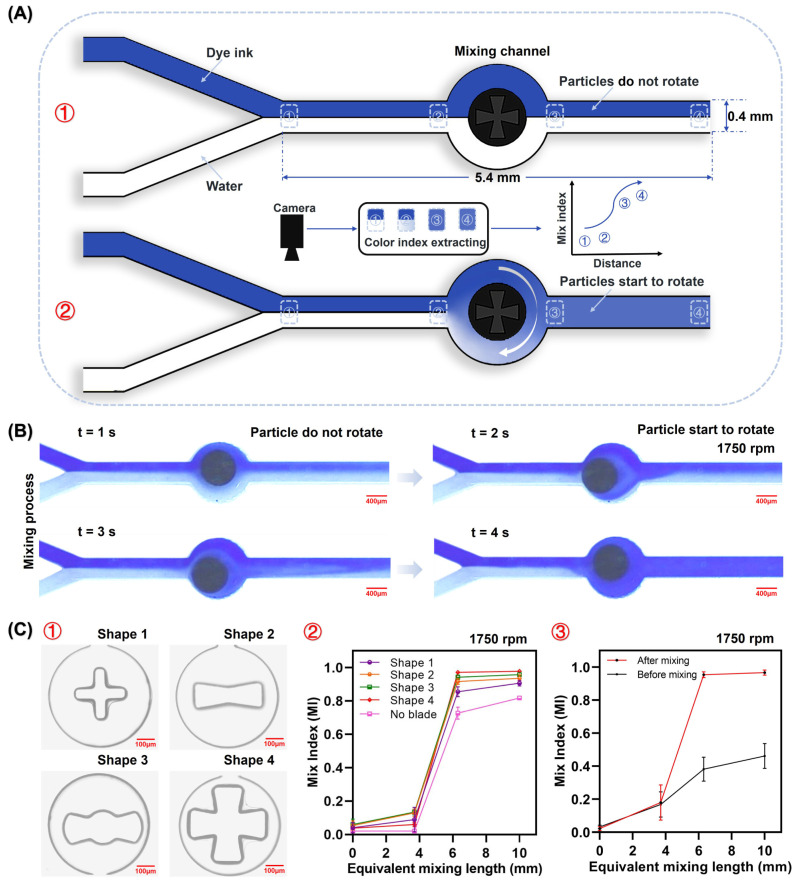
Controlled particle rotation for efficient mixing of different fluids. (**A**) Schematic diagram of the mixing channel and mixing experiment: ① Ink and water were not mixed when the particles were not rotated. ② The two fluids were mixed when the particles were rotated, and the pixel information in the four regions was captured and analyzed with a video camera. (**B**) Mixing processes guided by particles as active mixers. (**C**) The mixing effects of different shapes of particles in the four regions: ① Four different fan blades of particles were fabricated using microwells for mixing experiments. ② Mix indexes of the five different shapes of particles under the same conditions. ③ Before and after mixing of particles of shape 4 at different Reynolds numbers.

To quantitatively analyze the mixing performance of magnetic particles, blue dye ink and water were injected into the Y-shaped PDMS channel (Figure 5A), and we monitored the mixing process with a microscope and camera. We photographed four regions in the Y-shaped channel. The color information of each pixel in these four regions was analyzed and converted into a mixing index using Equation (1). Here, I_k_ is the intensity of the kth pixel, Ī_0_ is the average intensity, N is the total number of pixels, and MI is the ratio of the standard deviation of the intensities to the mean [42]. Because the intensity of the pixel is not zero when unmixed, the MI cannot be one in the fully mixed condition because of the backlight. Thus, Equation (2) [10] was applied to normalize the calculated mixing index, where MI_n_ is the normalized MI, MI_k_ is the MI calculated by Equation (1), MI_max_ is the maximum MI, and MI_min_ is the minimum MI. For the normalized MI, 0 denotes unmixed, while 1 denotes complete mixing.

(1) $$\mathrm{MI}=1-\frac{\sqrt{\frac{1}{N}\sum_{k=1}^{k=N}{\left(I_{k}-{\bar{I}}_{0}\right)}^{2}}}{{\bar{I}}_{0}}$$

(2) $${\mathrm{MI}}_{n}=\frac{{\mathrm{MI}}_{k}-{\mathrm{MI}}_{\min}}{{\mathrm{MI}}_{\max}-{\mathrm{MI}}_{\min}}$$

Figure 5B depicts the entire mixing process of a fluid when using particles as active mixers. The particles may be triggered within four seconds and mix with the fluid, and the entire mixing process is reversible, allowing for instant initiation. Previous studies [43] have referred and fabricated particles of different shapes and tested their liquid mixing ability (Figure 5C①). Disk-shaped particles with four different fan blades were placed in a Y-shaped PDMS channel, and two-phase fluids were introduced to observe the mixing effect under the action of magnetic particles of different shapes. In 12 repetitions of the experiment, particle No. 4 demonstrated the best mixing potential, achieving a mixing index of 97% for dye ink after rotational agitation, while the agitation of the other three particle shapes increased the mix index of the dye ink to more than 85%. This demonstrates that particles with specific angles and shapes increase the contact area with the fluid while improving the mixing efficiency. In addition, the particles without fan blades were also able to mix the fluid with a mixing index of 73%, and the presence of fan blades could further enhance the mixing efficiency (12% to 24%) of the particles [as indicated in Figure S3]. Following the mixing action of Particle No. 4, the mixing indexes of the nine fluid groups with different Reynolds numbers were all greater than 90%, with an average value 57% higher than before mixing (Region 3). Figure 5C③ statistically depicts the influence before and after mixing at various Reynolds numbers. It can be observed that mixing without particle intervention was inefficient and unstable. In contrast, the presence of particles significantly improved the fluid mixing efficiency and kept the mixing effects stable.

**Figure 6:**
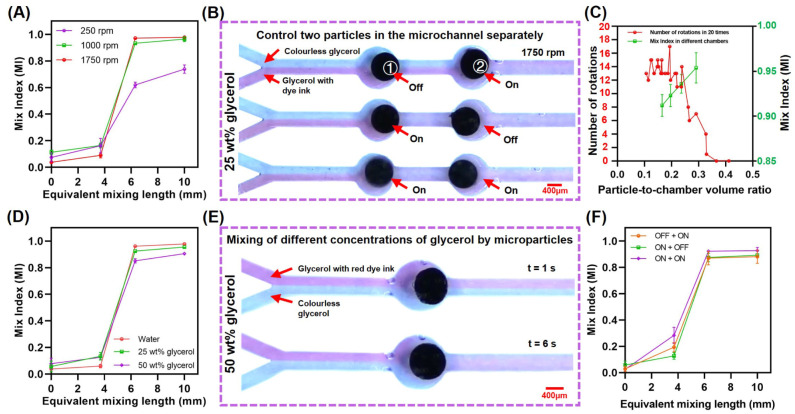
Mixing behaviors of particles in high-viscosity fluids. (**A**) Mix index of particles at different rotational speeds (36 repetitions of the experiment). (**B**) Control of the rotation of the two particles in the microchannel separately. (**C**) Particle rotation and mixing efficiency in chambers of different volumes. (**D**,**E**) Mixing results of particles at different concentrations of glycerol. (**F**) Simultaneous rotation of two particles improved mixing efficiency for highly viscous fluids.

The mixing efficiency of particles at different rotation speeds was recorded using a high-speed camera. The results indicated that when the rotation speed was below 1000 rpm, the mixing index increased significantly with the rotation speed increase. In addition, the increase in rotation speed became less significant with respect to the mixing index when the speed was over 1000 rpm (Figure 6A). As illustrated in Figure 6B, the rotation of two particles in the channel could be separately controlled, coupled with the extremely short start-up time of the particles (within 4 s), enabling us to carry out mixing missions at specific locations within the microchannel at specific time. The number of rotations of particles in different volume chambers in 20 experiments had been counted, and we calculated the mixing indices of the particles in four different volume chambers (Figure 6C). Larger chambers, although favorable for particle rotation, also slightly reduced the mixing efficiency. Placing two larger chambers in the same channel and rotating the particles inside them at the same time solved this contradiction skillfully.

In order to prove that magnetic particles can mix high-viscosity fluids, glycerol containing red dye ink and colorless glycerol were used as test samples, introduced into the Y-channel, and then analyzed regarding the mixing index in the same way, and the results (36 repetitions of the experiment) are shown in Figure 6D. The particles could complete the mixing task even in a high-viscosity medium (25 wt% and 50 wt% glycerol), and the mixing indices of both 25 wt% and 50 wt% glycerol exceeded 85%, which demonstrates the excellent performance of the particles in high-viscosity media. Additionally, in order to further enhance the mixing efficiency for high-viscosity fluids, two magnetic particles were placed in the same channel, and their mixing efficiencies were calculated under different rotation scenarios. Figure 6F shows that the mixing index of two particles rotating simultaneously could be improved by 6–12% compared to a single particle rotation (Region 3). It is worth mentioning that the system also mixes well with fluorescent microbeads (particle size of 14 microns, which is comparable to the size of a cell), which suggests a wider potential for biomedical applications [as indicated in Figure S5].

## 4. Conclusions and Outlook

In this study, a method for creating magnetic particles with quasi-three-dimensional shapes and employing them as active mixers to efficiently mix diverse fluids has been presented. Unlike mixing between two-phase laminar flows solely based on diffusion, the magnetic particles are driven by a magnetic field to rotate, dramatically improving the homogeneity and efficiency of sample mixing. This approach employs a degassed PDMS structure to provide self-priming power during particle production, enabling the automatic suction of a UV-curable adhesive suspension containing magnetic powder into the chip without the need for an external pumping component. The self-suction process simultaneously achieves self-dispensing of the samples. Subsequently, air separation is used to remove surplus UV-curable adhesive, which not only guaranteed particle integrity and high fidelity but also allowed us to manufacture hundreds of magnetic particles in less than 10 min.

The mixing performance of magnetic particles at various Reynolds numbers has been characterized and studied, as well as the mixing effects of particles with different shapes, with a mixing index ranging from 85% to 97%. Furthermore, the mixing performance of particles in glycerol has been measured, demonstrating their excellent mixing performance even in high-viscosity media. It was shown that the rotational speeds of the particles varied with the fluid viscosity, which gives them potential as fluid viscosity sensors. Notably, multiple magnetic particles can be placed in the same microchannel, allowing for individual control over their rotation. Additionally, the extremely short start-up time required for particle rotation facilitates on-demand mixing (efficient mixing at a specific time and locations) within microchannels. It has been reported that moving microrods and particles are capable of pumping fluids [44,45]. Our research, however, demonstrates that magnetic particles can rotate and mix fluids under magnetic field control, making them a promising candidate for use as micropumps and cell sorters. Furthermore, the particles could move in a certain direction along an external magnetic field, and numerous magnetic particles attracted and aligned with each other, implying their potential for applications in self-assembling robotics and drug delivery systems.

## Data Availability

The data that support the findings of this study are available from the corresponding authors upon reasonable request.

## Supplementary Materials

The following supporting information can be downloaded at https://www.mdpi.com/article/10.3390/bios14090408/s1: Table S1: UV-curable adhesive with different concentrations of magnetic powder. Figure S1: The process of fabricating bilayer particles using a chip consists of the following steps: (a) The first layer master mold is fabricated using mask A and photoresist. (b) The first layer of the master mold is left on the wafer after standard photolithographic follow-up. (c) Spin-coating photoresist on the first layer of the master mold. (d) The second layer of the structure is photolithographed on the first layer of the master mold using mask B and photoresist. (e) The second layer of the master mold is left on the first layer after standard photolithographic follow-up. (f–i) Fabrication of the PDMS chip and output of the particles. Figure S2: Schematic representation of the channels of the microstructural layer in the chip and the double-layer chamber. Figure S3: Mixing results for particles with and without fan blades under the same conditions. Figure S4: The use of refined magnetic powders improves the efficiency of particle yield. Figure S5: The mixing system is capable of mixing microbeads.
