# Supplementary material for: Fabrication of Patterned Magnetic Particles in Microchannels and Their Application in Micromixers
Source: Biosensors (Basel). 2024 Aug 23;14(9):408. doi: 10.3390/bios14090408 (PMC11430103; doi:10.3390/bios14090408)
Supplement: Supplementary file 1 [file biosensors-14-00408-s001.zip › biosensors-3143397-supplementary.pdf]

biosensors

MDPI

Supplementary Material

Fabrication of Patterned Magnetic Particles in Microchannels and Their Application in Micromixers

Tianhao Li †, Chen Yang †, Zihao Shao, Ya Chen, Jiahui Zheng, Jun Yang * and Ning Hu *

Key Laboratory of Biorheological Science and Technology, Ministry of Education and Bioengineering College, Chongqing University, Chongqing 400044, China; tianhaoli@stu.cqu.edu.cn (T.L.); yangchencq@cqu.edu.cn (C.Y.); spoonzh@stu.cqu.edu.cn (Z.S.); 202319131165@stu.cqu.edu.cn (Y.C.); jiahuizheng@cqu.edu.cn (J.Z.)
* Correspondence: bioyangjun@cqu.edu.cn (J.Y.); huning@cqu.edu.cn (N.H.)
† These authors contributed equally to this work.

Table S1. UV- curable adhesive with different concentrations of magnetic powder.

Concentrations of magnetic nanopowder	Solutes	Solvents
**0.05g/ml**	**Fe_3O_4 nanopowder**	**LOCTITE AA 3311**
**0.10g/ml**	**Fe_3O_4 nanopowder**	**LOCTITE AA 3311**
**0.15g/ml**	**Fe_3O_4 nanopowder**	**LOCTITE AA 3311**
**0.20g/ml**	**Fe_3O_4 nanopowder**	**LOCTITE AA 3311**
**0.25g/ml**	**Fe_3O_4 nanopowder**	**LOCTITE AA 3311**
**0.30g/ml**	**Fe_3O_4 nanopowder**	**LOCTITE AA 3311**
**0.35g/ml**	**Fe_3O_4 nanopowder**	**LOCTITE AA 3311**
**0.40g/ml**	**Fe_3O_4 nanopowder**	**LOCTITE AA 3311**

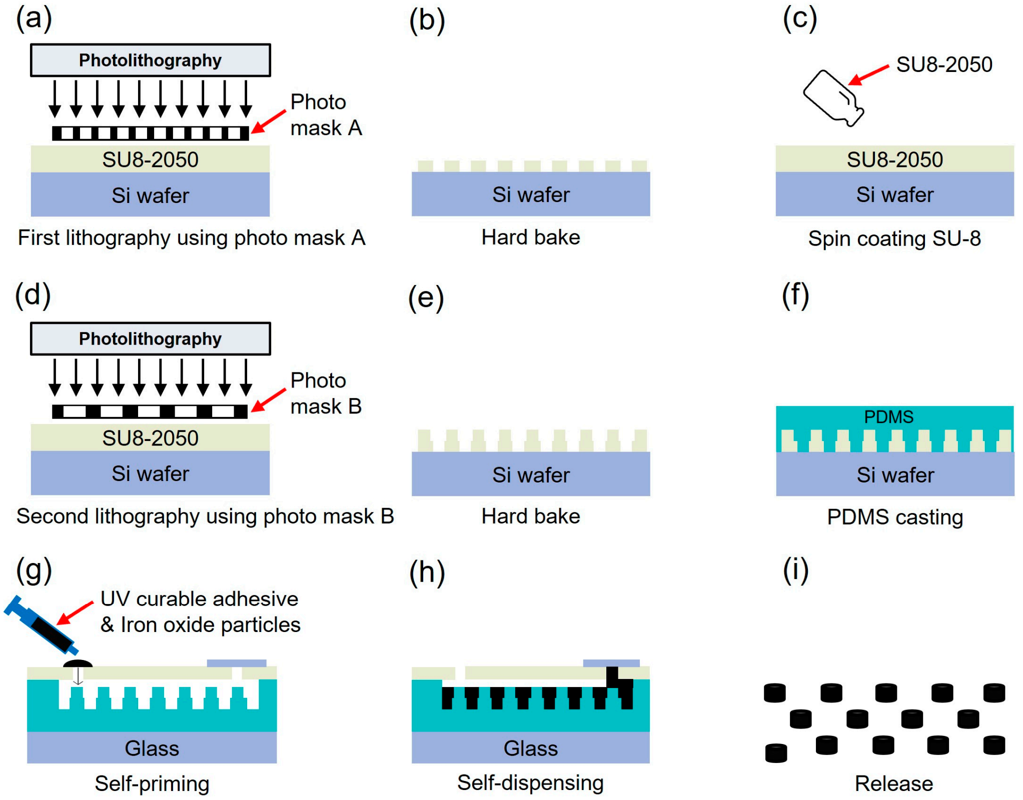

Figure S1. The process of fabricating bilayer particles using a chip consists of the following steps: (a) The first layer master mold is fabricated using mask A and photoresist. (b) The first layer of the master mold is left on the wafer after standard photolithographic follow-up. (c) Spin-coating photoresist on the first layer of the master mold. (d) The second layer of the structure is photolithographed on the first layer of the master mold using mask B and photoresist. (e) The second layer of the master mold is left on the first layer after standard photolithographic follow-up. (f-i) Fabrication of the PDMS chip and output of the particles.

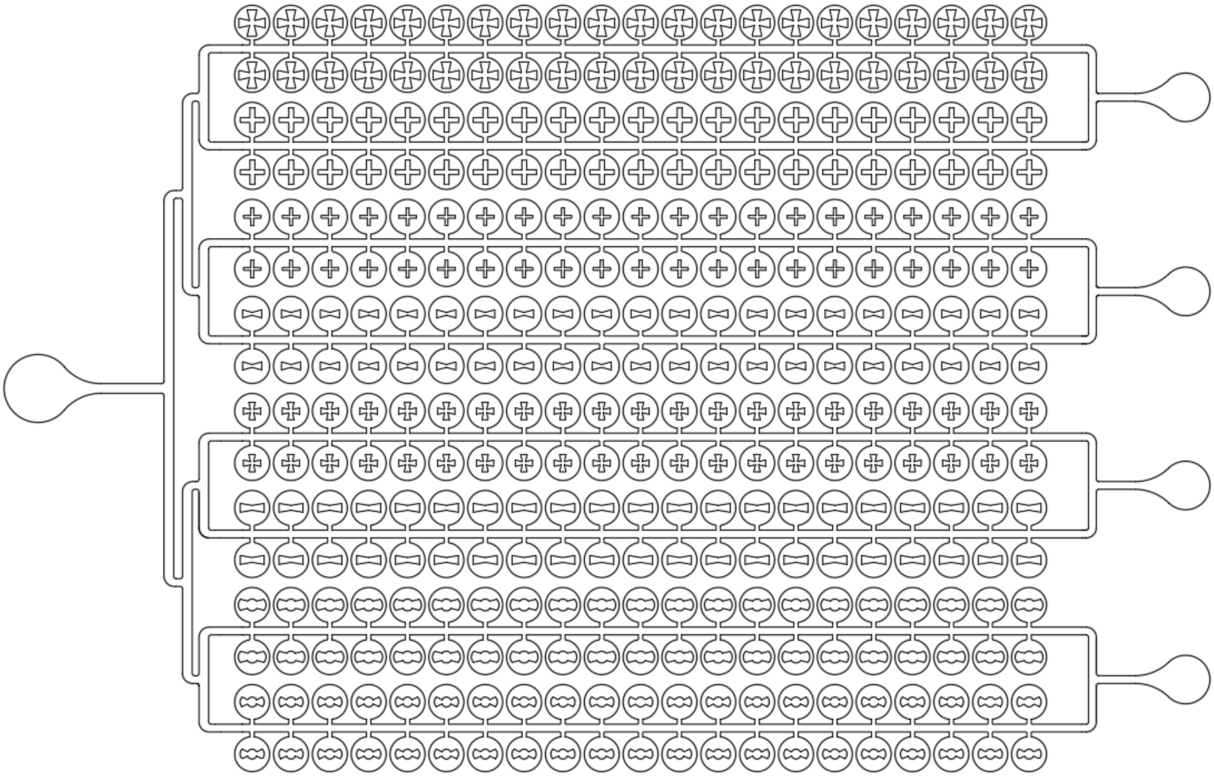

Figure S2. Schematic representation of the channels of the microstructural layer in the chip and the double-layer chamber

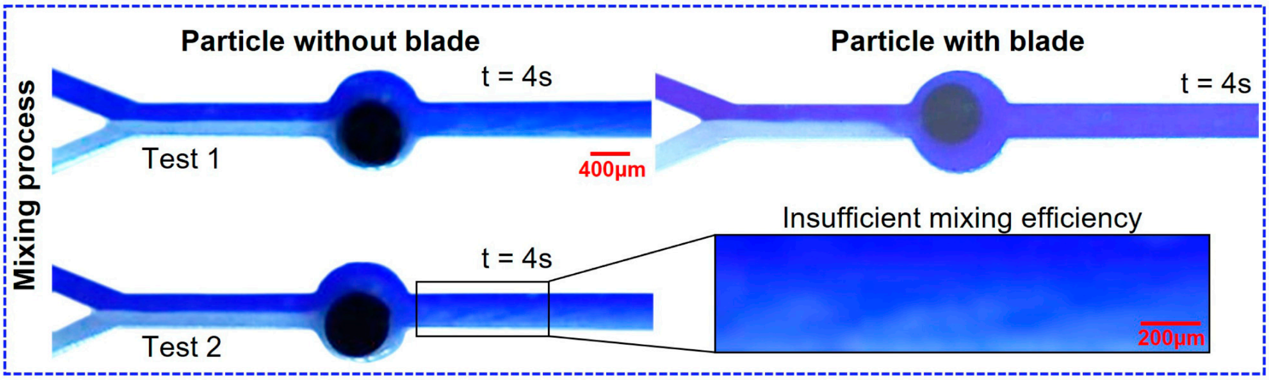

Figure S3. Mixing results for particles with and without fan blades under the same conditions

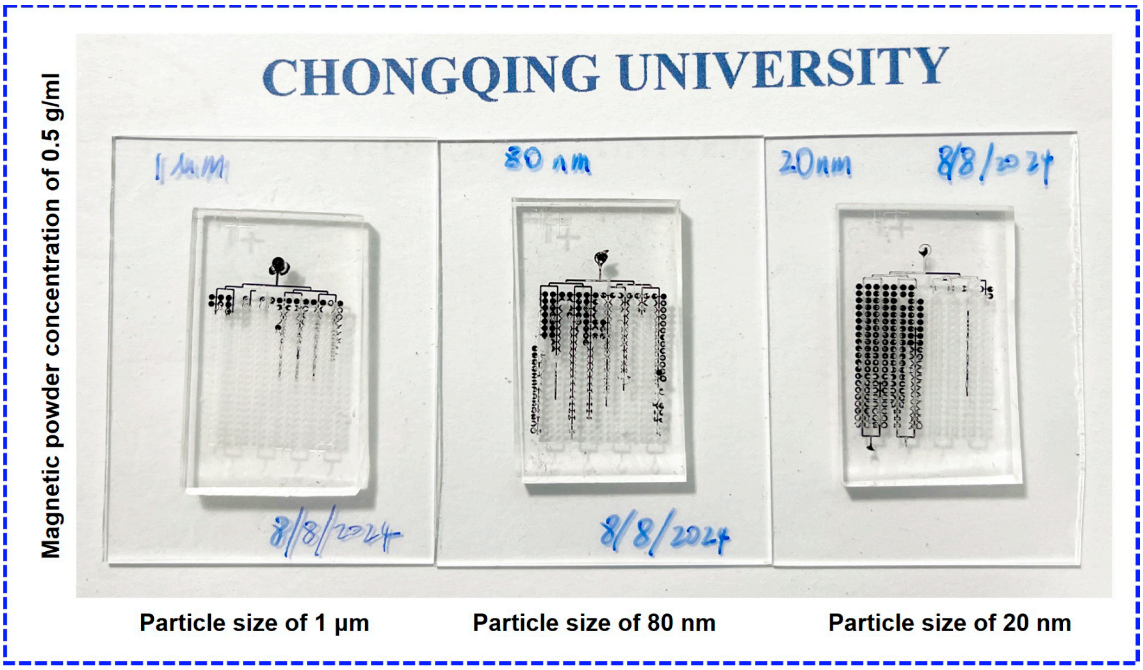

Figure S4. The use of refined magnetic powders improves the efficiency of particle yield.

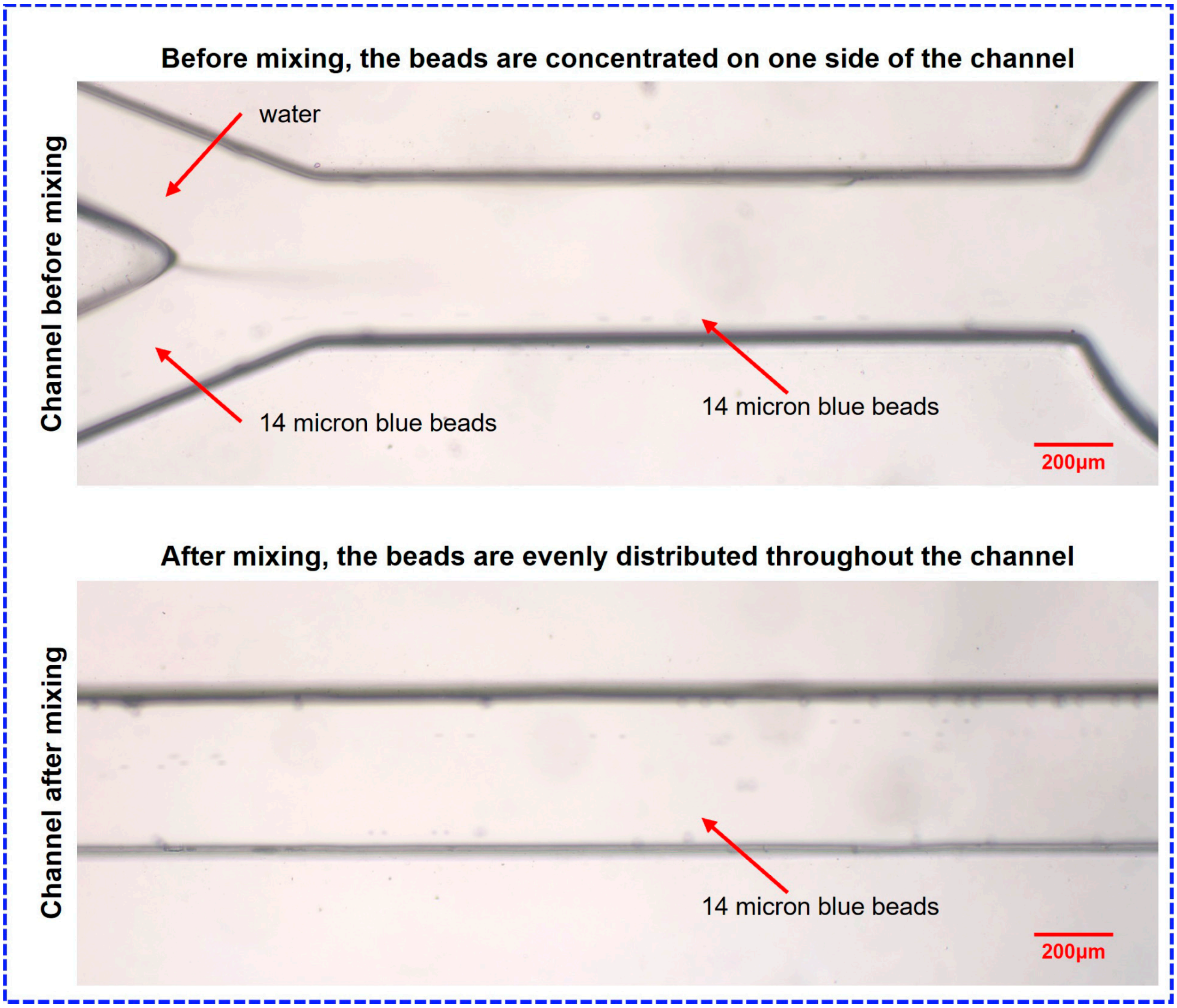

Figure S5. The mixing system is capable of mixing microbeads
